# When the Rule Becomes the Exception. No Evidence of Gene Flow between Two *Zerynthia* Cryptic Butterflies Suggests the Emergence of a New Model Group

**DOI:** 10.1371/journal.pone.0065746

**Published:** 2013-06-06

**Authors:** Francesca Zinetti, Leonardo Dapporto, Alessio Vovlas, Guido Chelazzi, Simona Bonelli, Emilio Balletto, Claudio Ciofi

**Affiliations:** 1 Dipartimento di Biologia, Università degli Studi di Firenze, Florence, Italy; 2 Istituto Comprensivo Materna Elementare Media Convenevole da Prato, Prato, Italy; 3 Dipartimento di Scienze della Vita e Biologia dei Sistemi, Università degli Studi di Torino, Torino, Italy; Ben-Gurion University of the Negev, Israel

## Abstract

There is increasing evidence that most parapatric cryptic/sister taxa are reproductively compatible across their areas of contact. Consequently, the biological species concept, which assumes absence of interbreeding, is becoming a not so effective criterion in evolutionary ecology. Nevertheless, the few parapatric sister taxa showing complete reproductive barriers represent interesting models to study speciation processes and the evolution of reproductive isolation. In this study, we examined contact populations in northwestern Italy of two butterfly species, *Zerynthia polyxena* and *Z. cassandra*, characterized by different genitalic morphotypes. We studied levels of divergence among 21 populations distributed from Sicily to France using three genetic markers (the mitochondrial COI and ND1 genes and the nuclear wingless gene) and genitalic geometric morphometrics. Moreover, we performed species distribution modelling to estimate different climatic requirements of *Z. polyxena* and *Z. cassandra*. We projected climatic data into glacial maximum scenarios in order to verify if and to which extent glacial cycles could have contributed to speciation processes. Genetic and morphometric analyses identified two main groups. All specimens showed a concordant pattern of diversification, including those individuals sampled in the contact area. Haplotype distribution and climatic models showed that during glacial maxima both species experienced a strong range contraction and presumably remained separated into different microrefugia in southern France, in the Italian Peninsula and on the islands of Elba and Sicily. Long term separation was probably favoured by reduced dispersal ability and high phylopatry, while genitalic diversification probably favoured interbreeding avoidance. Conversely, the aposematic wing pattern remained almost identical. We compared our results with those obtained in other species and concluded that *Z. polyxena* and *Z. cassandra* represent a valuable model in the study of speciation.

## Introduction

In biparental species, gene exchange across populations is strictly linked to mating and to production of fertile offspring. For this reason, the biological concept, identifying species as reproductively isolated interbreeding groups of individuals [1] received large support by ecologists and evolutionary biologists. However, molecular studies have shown that hybridization and introgression among well established species are relatively common events, mainly in parapatric sister taxa at their contact zones (e.g. [2], [3], [4], [5], [6]). Increasing evidence for a grey-scaled species delimitation made the assumption of complete reproductive isolation weaker and new species concepts were then introduced (reviewed by [7]). Recent theories suggest that species may even differentiate in the presence of hybridization, provided that some genes linked to differential fitness or to sexual interactions are exchanged at low frequencies [7], [8], [9], [10], [11]. In butterflies, on the other hand, adaptive genes can be exchanged among species million of years after speciation events [6].

Contact zones between sister and cryptic species represent excellent models for the study of speciation, particularly when species showing complete reproductive isolation and/or highly permeable reproductive barriers can be compared [4]. European butterflies are often used as model organisms for such studies [4], [12], [13]. However, comparative research has been hampered by the high proportion of sister taxa showing weak reproductive barriers. A recent revision of the entire European butterfly fauna has shown that 16% of 440 species hybridize in nature, often producing fertile hybrids, and that parapatric sister species are almost always involved in this process [4].

There are exceptions to this pattern. The reproductively isolated cryptic sister taxa of the *Leptidea* genus, for instance, are characterized by identical appearance and relatively low genetic divergence [12], [13], [14]. Complete reproductive isolation reached in a relatively short time (between 270,000 and 120,000 years) in the absence of divergent external traits [12], [13] has been considered a “perfect crime” by Descimon & Mallet [4] and represents an excellent context to study butterfly evolution [4], [11], [13]. Based on analysis of male genitalic structures, Dapporto [15] identified two completely distinct morphotypes in the broadly recognized species of the Papilionid butterfly *Zerynthia polyxena*. One morphotype is segregated to the Italian Appennines (*Z. cassandra*) while the other is found in remnant southern and central European populations (*Z. polyxena*). These morphotypes come into close contact in north-western Italy, where they also occur in sympatry in the Beigua mountain area with no evidence of morphologically intermediate individuals [15]. Genetic data are scarce, only two specimens from the *Z. cassandra* range were sequenced for the mitochondrial COI gene and no information was provided on their genitalic characters [16]. However, these specimens revealed about 2% divergence with respect to other European individuals.

In this study, we assessed levels of genetic divergence and introgression at the contact zone between *Z. cassandra* and *Z. polyxena* by integrating geometric morphometric comparison of male genitalia and sequence analysis of one nuclear and two mitochondrial DNA markers. Moreover, we applied distribution modelling to assess species occurrence in different climatic settings across the current range. We then projected such differences into reconstructed climatic scenarios of the last glacial period and evaluated the extent to which distributional changes driven by climatic events may have been responsible for long term genetic isolation. Considering the clear differences in genitalic morphology, identical aposematic wing colouration and well defined contact area between *Z. cassandra* and *Z. polyxena*, new evidence on genetic variation and climatic preferences may indicate whether these species are likely to be reproductively separated and which factors shaped the observed distributional patterns. Our dataset may also suggest whether the sister taxa examined in this work represent a good model for butterfly evolutionary studies.

## Materials and Methods

### Geometric Morphometrics

Geometric morphometrics was conducted on 217 *Zerynthia polyxena* and *Z. cassandra* males from Italy and southern France. Samples included 186 individuals from a dataset published by Dapporto [15]. Permission to collect species listed under the 92/43/EEC Annex IV was granted by the Italian Ministry of the Environment (U.prot PNM-2011-0010400-13/05/2011). Additional specimens were obtained from public Museums and Research Institutions (Table S1). All specimens are deposited in public collections. Of particular relevance are 15 males collected in the area of sympatry on Mount Beigua, where three *Z. polyxena* were collected in 1997, four *Z. polyxena* and two *Z. cassandra* in 1999, three *Z. cassandra* in 2000 and two *Z. cassandra* and one *Z. polyxena* in 2009. Landmarks and sliding semi-landmarks [17] were identified on the lateral section of the valvae using the TPS (thin-plate spline) software. Six points along the valvae rim were considered as landmarks, while 24 additional points were defined as sliding semi-landmarks which could slide along the outline trajectory ([17] and Figure S1). Digitalization and definition of sliders were carried out using TPSDIG 2.16 [18] and TPSUTIL 1.53 [19], respectively. A generalized procrustes analysis was applied to landmark data in order to remove variation in location, scale and orientation, and to superimpose the objects into a common coordinate system [17]. We calculated partial warps from the shape residuals of the generalized procrustes analysis using TPSRELW 1.49 [20]. We then obtained principal components (PCs or relative warps) by applying a principal components analysis. We used the relative warps in a partitioning around medioids (PAM) analysis implemented in the *cluster* R package in order to identify the most likely number of morphotypes in our sample as suggested by Borcard et al. [21]. We generated specimen clusters between 2 and 216 and selected the partition showing the highest silhouette width (a relative measure of inter- versus intra-clusters dissimilarity spread) as the most suitable number of morphotypes.

### Genetic Analysis

A subset of 106 *Z. polyxena* and *Z. cassandra* individuals were used for DNA analysis (Table S1). DNA was extracted using standard phenol-chloroform procedures. Two mitochondrial and one nuclear genes were sequenced and compared. The full-length “barcode” region (657 bp) of the mitochondrial cytochrome oxidase I (COI) gene was amplified and sequenced using the light-strand primer Parn_Zer_F1439 (5′ - ATCGCTTATACTCAGCCATC - 3′) and the heavy-strand primer Parn_Zer_R2185 (5′ - GGGAAATTATTCCAAATCCTG - 3′) designed on the tyrosine tRNA and the COI gene, respectively, using a consensus sequence of *Z. polyxena* and *Parnassius bremeri* COI genes [22], [23]. Primer numbers refer to the 3′ end of the published *Parnassius bremeri* mitochondrial genome sequence [23]. A 472 bp fragment of the mitochondrial NADH dehydrogenase subunit 1 (ND1) gene was amplified and sequenced using PCR primers described in [24]. Finally, amplification and sequence of a 393 bp fragment of the nuclear DNA wingless (wg) gene was obtained using the light-strand primer wg_F29 (5′ - CAGTAAAGACTTGCTGGATGC - 3′) and the heavy-strand primer wg_R382 (5′ - TGCACCTTTCAACCACAAAC - 3′) designed specifically for this project. The 3′ base of these primers refer to the published *Zerynthia polyxena* partial wingless sequence [22].

Polymerase chain reaction (PCR) amplifications were conducted in a total volume of 25 µl containing 1–5 µl of extracted DNA, 1× PCR buffer, 1.5 mM MgCl_2_, 100 µM of each dNTP, 0.5 µM of each primer and 1 unit of *Taq* DNA polymerase (Invitrogen). Thermal profiles for COI amplification consisted of an initial denaturation step of 5 min at 94°C, followed by 35 cycles of 30 sec at 94°C, 30 sec at 50 and 90 sec at 72°C, with a final extension step of 7 min at 72°C. PCR profiles for ND1 and wg amplification had an initial denaturation step of 5 min at 94°C, followed by 35 cycles of 30 sec at 94°C, 30 sec at 52°C and 60 sec at 72°C, with a final extension step of 7 min at 72°C. Samples that resulted in a poor PCR product were amplified using 1 µl of extracted DNA, 1× Restorase buffer, 200 µM of each dNTP, 0.5 µM of each primer and 1.25 units of Restorase DNA polymerase (Sigma-Aldrich), a blend of high quality *Taq* DNA polymerase and a DNA repair enzyme which has proved effective in the amplification of damaged DNA [25]. PCR mix was incubated for 15 min at 37°C and for 5 min at 72°C. Primers were then added to the mix prior to amplification. PCR cycles consisted of an initial denaturation step of 2 min at 94°C, followed by 40 cycles of 30 sec at 94°C, 30 sec at 50–52°C and 60 sec at 72°C, with a final extension step of 5 min at 72°C. PCR products were cycle-sequenced using BigDye Terminator v3.1 (Applied Biosystems) according to the manufacturer’s protocol. Cycle sequencing reactions were resolved on an Applied Biosystems 3100 DNA analyzer and raw sequence chromatographs from both strands were edited and aligned using CodonCode Aligner 3.0.1 (CodonCode Corporation). The resulting consensus sequences consisted of a total of 657, 438 and 348 nucleotides of COI, ND1 and wg genes, respectively (Genbank accession numbers: KC119707– KC119746).

### Haplotype Networks and Phylogenetic Analysis

Sequences were checked for insertions, deletions and stop codons that would result in non-functional proteins. Mean maximum likelihood (ML) distances within and between species and among localities were calculated using MEGA 5 [26]. We inferred haplotypes networks for COI and ND1 genes using the median-joining network method [27] implemented in the program NETWORK 4.6.1.0 (http://www.fluxus-engineering.com). We set the same default weight (10) for each character/site, given the absence of hyper-variable sites. The Epsilon parameter was set to 0. We used the default "connection cost" distance calculation methods and we applied the MP option [28] on results of the median joining calculation. Eight GenBank sequences of *Z. polyxena* from France, Romania and Russia [22], [29], [30] were also used for construction of haplotype networks.

Best fit of molecular evolution model to each of our dataset was assessed using JMODELTEST [31] under the Bayesian Information Criterion. Likelihood values were calculated for 88 models using a maximum likelihood optimization of tree topology implemented in Phyml [32]. Each model was tested allowing for variation in nucleotide frequencies, different substitution rates among sites and proportion of invariable sites. The models of sequence evolution that best fit the COI and ND1 datasets were the Kishino and Yano (HKY) model [33] and the Transition model (TIM) [34], respectively. The Kimura 2-parameter (K80) model [35] resulted the best fit for the wingless dataset.

Partitioned Bayesian inference was applied to the combined dataset of COI, ND1 and wg sequences. Bayesian inference was conducted using Metropolis-coupled Markov chain Monte Carlo method implemented in MRBAYES 3.1.2 [36] applying the best substitution model for each partition of the combined dataset. The TIM and the K80 models could not be implemented in MRBAYES. We applied the general time-reversible model (GTR) [37], which best matches the assumptions of the transition model, and the HKY model (instead of the K80 model) with equal stationary state frequency. Approximation of the posterior probabilities of trees was performed by two independent runs starting with default prior values and initial random trees with three heated and one cold Markov chains, which ran for 2×10^6^ generations with a 1000 generation sampling interval. Stationarity of the analysis was determined by examining the standard deviation of split frequencies between the two simultaneous runs and the potential scale reduction factor [38]. The first 25% of trees were discarded as burn-in and trees were used for analysis only after the chain became stable. The remaining trees were used to construct a 50%-majority rule consensus tree. GenBank sequences of the Papilionidae *Zerynthia rumina*, *Bhutanitis thaidina* and *Allancastria cretica* [22], [30] were used as outgroups. The phylogenetic tree was rooted on *Bhutanitis thaidina*.

### Distribution Modelling

Locations of 334 European occurrence sites for *Z. cassandra* and 575 sites for *Z. polyxena* were assessed based on known species distribution [15], [39], (Dapporto & Zinetti unpublished data). The 19 WorldClim variables [40] were used to perform maximum entropy distribution modelling. We used a resolution grid of 0.04 degrees. These variables generally show collinearity which may bias results. We therefore selected 10,000 random cells across a plot in the Mediterranean region spanning from 1°00′00″ E to 35°00′00″ E and from 33°00′00″ N to 70°00′00″ N. For each cell, we analyzed the correlation among the WorldClim variables. We then selected the most biologically meaningful variable for those cases in which two or more variables showed a Pearson correlation coefficient higher than 0.8. Biological variables were selected mostly on the known species requirements based on adults activity and/or extreme climatic conditions experienced by larvae. We predicted potential distributions based on butterfly presence data using MAXENT 3.3.2 (http://www.cs.princeton.edu/~schapire/maxent). The software implements a machine-learning algorithm based on maximum entropy to identify areas with optimal environmental conditions [41]. Considering that different sampling efforts for different areas can produce false signals of climatic preferences [41], we applied a spatial filter to select a single random specimen for each cell of 0.4×0.4 degrees using the gridSample function implemented in the R package *DISMO*. The filter resulted in 117 and 287 presence points for *Z. cassandra* and *Z. polyxena*, respectively. We used default parameter settings and removed hinge and likelihood features to increase prediction accuracy [42]. Each model was replicated 100 times with a cross-validation test performed on 10% of presence data. Goodness of the model was examined using receiver operating characteristic (ROC) plots and quantified by the area under curve (AUC). AUC values higher than 0.7 indicated that predictions of the model were higher than random values [43]. Relative importance of variable contribution was assessed by Jackknife of sequential variables removal [41]. We assessed models for butterfly distributions in modern day climate and then projected expected distributions into Last Glacial Maximum (LGM) WorldClim data. We used the Community Climate System Model (CCSM) and the Model for Interdisciplinary Research on Climate (MIROC) obtained from the Paleoclimate Modelling Intercomparison Project Phase II using the same variables of the modern climate models. These models differ in the reconstruction of several climatic variables and are well known to produce different results. For instance, CCSM and MIROC tend to overestimate and underestimate, respectively, proxy evidence of LGM winter sea ice [44]. As a result, for Mediterranean butterflies, the CCSM model tends to project narrower distributions at LGM than MIROC (e.g. [45], [46]).

## Results

### Geometric Morphometrics

**Figure 1:**
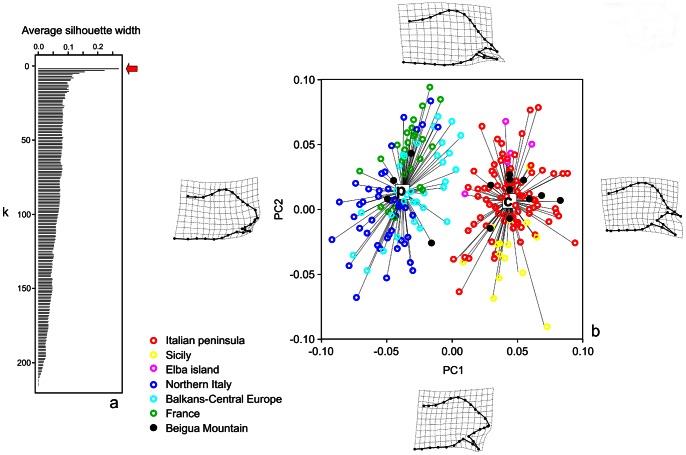
Analysis of genitalic morphology. a) Partitioning Around Medioids (PAM) analysis. The red arrow indicates the solution that assumes the existence of two morphotypes having the best fit (highest average silhouette width) among all possible partitions. b) Partition in two groups obtained by PAM analysis plotted for the first two relative warps (PC1 and PC2). A high concordance with geographic origin is shown for both *Z. cassandra* (c) and *Z. polyxena* (p) morphotypes with the only exception of the Mount Beigua population (black dots), which includes individuals belonging to both species. Deformation grids of male genitalia confirm previous results on morphological diversification between the studied species [15].

Analysis of 217 male genitalia resulted in 52 relative warps. PAM analysis showed that the partition in two clusters had the highest silhouette width (Fig. 1a). This result supported the distinction of two morphotypes. Specimen assignment by PAM confirmed that all samples originating from Italy, south to the river Po, grouped in the first cluster (*Z. cassandra*), while the other populations were included in the second cluster (*Z. polyxena*). Specimens from Mount Beigua were found in both groups (Fig. 1b). Shape variability along PC1 (35.16% of variance explained) and PC2 (22.63% of variance explained) indicated that most shape variance was explained by the length of the valvar apex (Fig. 1b).

### Genetic Diversity and Haplotype Networks

We obtained 103 consensus sequences and 22 haplotypes characterized by 35 variable nucleotide sites for the COI gene, and 86 sequences and 14 haplotypes defined by 23 polymorphic sites for the ND1 gene. Unambiguous sequences of the nuclear DNA wingless gene were obtained for 97 individuals. A total of four wg genotypes were characterized by four polymorphic sites. All *Z. cassandra* specimens showed the same wg genotype, while three different genotypes were found for *Z. polyxena.* Three samples of *Z. polyxena* were heterozygous at two polymorphic sites (Table 1). Maximum likelihood mean distances between species were consistently higher than within species values for COI, ND1 and wg genes (Table 2 and Table S1).

**Table 1:** Nucleotide variation and genotype designation in 97 *Zerynthia cassandra* and *Z. polyxena* individuals sequenced for the wingless (wg) gene.

wg genotype	Polymorphic site				Specimens
	57	132	165	237	
W1	A	G	C	G	*Z. cassandra* (*N* = 76)
W2	G	A	T	A	*Z. polyxena* (*N* = 17)
W3	G	A	C	G	*Z. polyxena* (*N* = 1)
W4	G	A	T/C	A/G	*Z. polyxena* (*N* = 3)

**Table 2:** Genetic diversity of COI, ND1 and wingless genes in *Zerynthia cassandra* and *Z. polyxena*.

	*Zerynthia cassandra*			*Zerynthia polyxena*				
	*N*	h	A+T	*N*	h	A+T	ML between species	ML within species
COI	79	12	0.71	24	10	0.71	0.014±0.005	0.006±0.002
ND1	75	10	0.77	11	4	0.78	0.032±0.008	0.011±0.003
wg	76	1	0.46	21	3	0.46	0.010±0.005	0.004±0.002

Number of individuals for which unambigous consensus sequences were obtained (*N*), number of haplotypes or genotypes (h) and A+T mean frequencies are shown for each species. Maximum likelihood (ML) mean distances between and within species are also reported.

**Figure 2:**
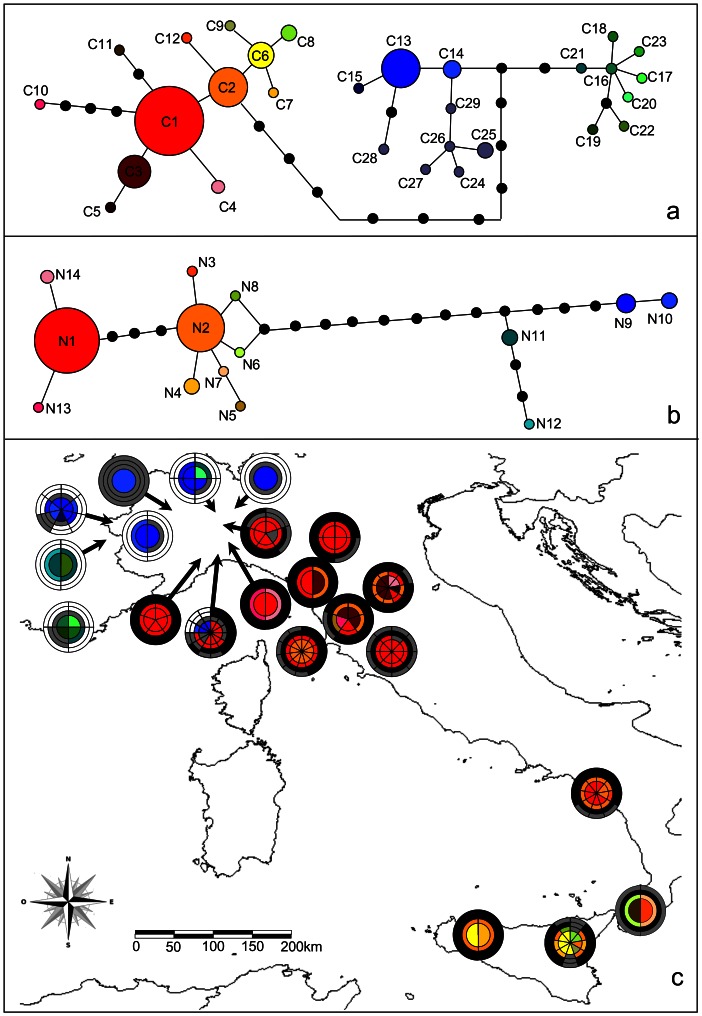
Median-joining haplotype network of mitochondrial DNA COI (a) and ND1 (b) sequences for *Zerynthia cassandra* and *Z. polyxena*. Eastern European haplotypes (C24–C29) are shown in the same bullet color. The lower map (c) shows distribution of haplotypes and genitalic morphotypes for each sampling location. Each pie is divided into a number of slices equal to the number of sampled butterflies for that location. Each slice is further divided into four sectors. Starting from the centre of the pie, the first two sectors show the COI and ND1 haplotype colors, respectively, found at that location (see networks reconstruction). The third and the outer sectors of the slice show assignment of wingless genotype and genitalic morphotype, respectively, to either *Z. cassandra* (black) or *Z. polyxena* (white). A grey sector indicates absence of the corresponding marker for that individual.

In the COI haplotype network, *Z. cassandra* and *Z. polyxena* were separated by a minimum of 13 mutations (from C2 to C14, Fig. 2a). C1 was the most common *Z. cassandra* haplotype in Italy and the only haplotype of this species occurring north of Tuscany. The second most common haplotype was found in southern Italy and was the only sequence occurring on Elba island. Other *Z. cassandra* haplotypes were found in central and southern Italy. The COI haplotype network for Z. *polyxena* described two major groups. The first one included haplotypes from France, while the second grouped sequences from eastern Europe and northern Italy (Fig. 2a). One haplotype from the French region was also found in northern Italy. The ND1 haplotype network revealed a similar pattern with haplotypes of *Z. cassandra* and *Z. polyxena* separated by a minimum of 10 mutations (from N6–N8 to N11, Fig. 2b). N1 was the most common *Z. cassandra* haplotype occurring in northern and central Italy. The second most common haplotype (N2) occurred from Tuscany down south to Sicily. The ND1 network confirmed the characterization of a French and a northern Italian haplogroups for *Z. polyxena*. The Italian specimen showing the French COI haplotype had also a French ND1 sequence. All specimens from France and the Italian Alpine region had a *Z. polyxena* haplotype, corroborating geometric morphometrics results (Fig. 2c). On the other hand, all individuals from the Apennines had a *Z. cassandra* haplotype for all markers, with the exception of specimens from Mount Beigua where haplotypes of both taxa were found in sympatry but segregated in accordance to morphometric analyses (Fig. 2c).

### Phylogenetic Analyses

**Figure 3:**
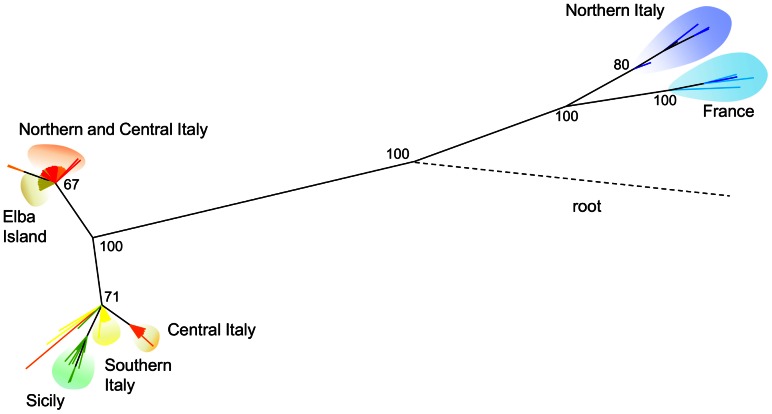
Majority rule (50%) consensus tree resulting from Bayesian analysis of the combined COI, ND1 and wingless gene datasets. Node supports inferred from Bayesian posterior probability are shown above recovered branches.

The topology of the Bayesian tree constructed using the combined genetic datasets revealed two monophyletic lineages, supported by 100% posterior probability values (Fig. 3). The first lineage included all specimens morphologically attributed to *Z. cassandra*, while the second lineage comprised all *Z. polyxena* samples. The first lineage was characterized by two main clades. The first one included specimens from central and southern Italy and Sicily. All but one individuals from Sicily were grouped together. The second clade comprised specimens from northern and central Italy (including those from the region of sympatry) and Elba Island. The lineage of *Z. polyxena* consisted of two well supported clades. The first clade included all specimens from France and one from northern Italy (Vercelli), while the second one consisted of all samples from northern Italy.

### Species Distribution Modelling

**Figure 4:**
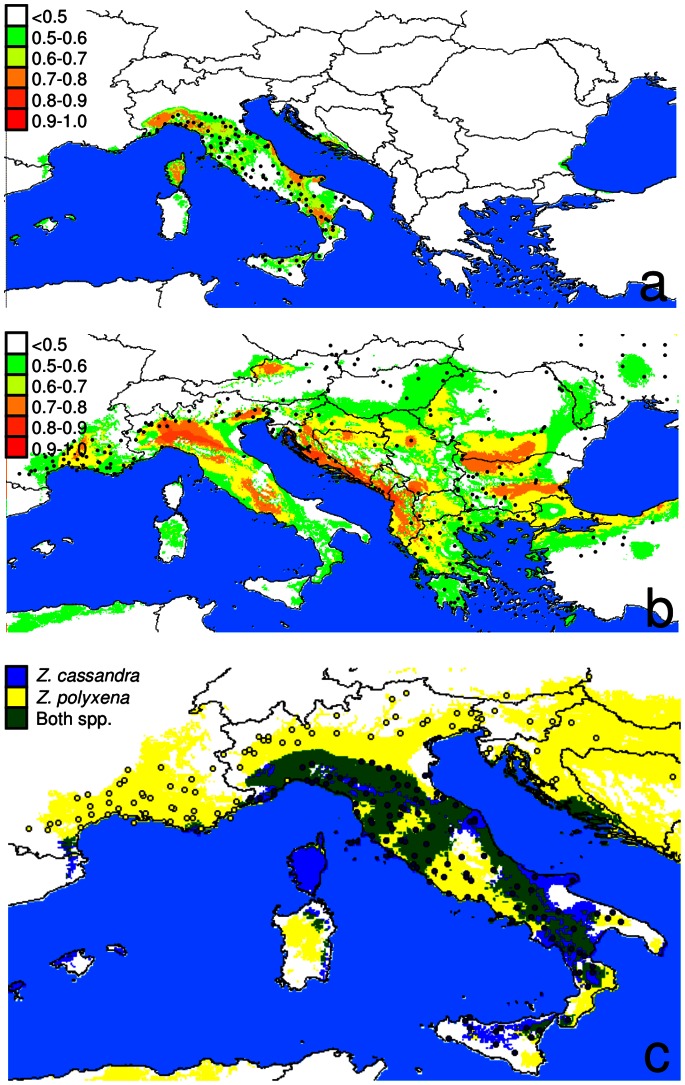
Representation of the logistic output of the Maxent analyses for *Zerynthia cassandra* (a) and *Z. polyxena* (b). Values >0.5 indicate the likely presence of a species. Bullets show current distributions. In the lower map (c) blue and yellow areas show logistic output >0.5 for *Z. cassandra* and *Z. polyxena*, respectively. Areas where both species are predicted to occur are reported in green. Bullet and circles show current distribution of *Z. cassandra* and *Z. polyxena,* respectively.

**Figure 5:**
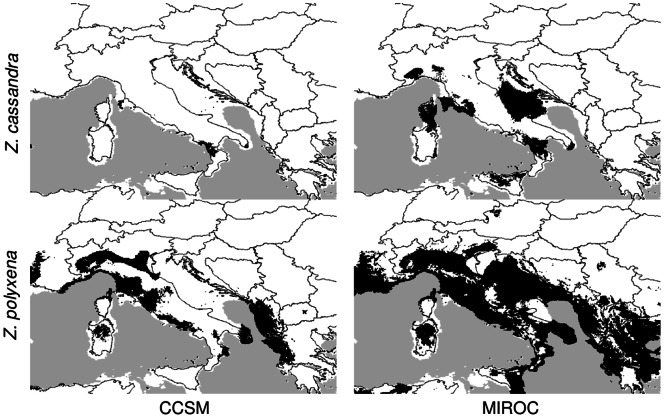
Projection of the Maxent models (based on present species distribution and climate data) on climatic reconstruction for the last glacial maximum using MIROC and CCSM circulation models. Dark areas show predicted species distribution (logistic output >0.5) during the last glacial age.

The MAXENT modelling resulted in a good fit for both species, with an AUC score of 0.861 and 0.977 for *Z. polyxena* and *Z. cassandra*, respectively. The Jackknife evaluation of the importance of variables revealed that temperature was more important than precipitation. Maximum temperature in the warmest month (BIO5), minimum temperature in the coldest month (BIO6), mean precipitation in the driest quarter (BIO9) and mean diurnal temperature range (BIO2) were most important in the *Z. polyxena* model (File S1), whereas BIO6, BIO9 and precipitation in the warmest quarter (BIO18) were significant in the *Z. cassandra* model (File S2). The areas with a logistic response higher than 0.5 largely matched the observed species distributions. The only exception was the Italian peninsula, which was predicted to have a suitable climate for *Z. polyxena* despite the total absence of the species from this area (Fig. 4). In particular, the regions predicted to be potential areas of distribution of the two species largely overlapped in the Apennines and, to a lesser extent, in the Maritime Alps and southern France. Projections of the two climatic reconstructions for the last glacial maximum revealed different results between the MIROC and the CCSM models. In particular, the MIROC model projected a larger area with logistic values higher than 0.5 (Fig. 4), a pattern found in other butterfly studies (e.g. [45], [46]) In these projections, both species showed a reduced expected occurrence in the northern regions of the Mediterranean range and a fragmented distribution in southern areas. However, in both the MIROC and CCSM models, *Z. polyxena* was expected to occur across the Italian peninsula (Fig. 5).

## Discussion

### Species Divergence

The Papilionidae *Zerynthia cassandra* and *Z. polyxena* revealed a strong and consistent pattern of genetic and morphological differentiation. Analysis of mitochondrial and nuclear DNA and genitalic morphometric data resulted in a phylogenetic reconstruction that clearly divided samples collected from Sicily to southern France into two clades. Divergence of traits was maintained in the region where *Z. cassandra* and *Z. polyxena* occur in sympatry, with no evidence for hybridization.

Levels of genetic divergence between *Z. cassandra* and *Z. polyxena* (1.5% COI, 3% ND1 and 1% wg) were similar to, or lower than, values reported for most European sister and/or cryptic species comparisons [13], [29], [47], [48], [49]. These studies also showed that most taxa hybridize at their contact areas. Hybrid zones are generally large (50–250 km) in butterfly species for which introgression occurs over their European range [13], [29], [49], [50], [51]. The cryptic butterflies *Polyommatus icarus/P. celina*, and *Aricia agestis/A. cramera*, for instance, have hybrid zones 200 and 50 km wide, respectively, and show clear phylogenetic divergence from 3% to 5% of COI sequences. Nevertheless, they show clear evidence of introgression with intermediate morphotypes and discrepancy between nuclear and mitochondrial DNA sequences [49], [52]. We sampled very close populations of *Z. cassandra* and *Z. polyxena* (Vercelli, Vigevano and Alessandria) with no apparent geographical barriers and analyzed specimens from the only area where the two species have been found in sympatry (Mount Beigua). We recorded no evidence of introgression of either mitochondrial, nuclear or morphological markers despite evidence of rather long dispersal was suggested by the occurrence of a French haplotype in a specimen collected in northern Italy. The Vigevano and Vercelli sampling sites of the northern Italy contact zone, where we found only *Z. polyxena* morphotypes and DNA sequences, are 37 and 50 km from Alessandria, respectively, where only *Z. cassandra* sequences and morphotypes are recorded. Although no evidence for introgression in a limited set of specimens and markers is not a definitive proof of lack of hybridization, we recovered a very different pattern from that described for most European sister taxa. Moreover, although a very narrow hybridization area may occur between these localities, the belt would be strikingly narrower than those reported for other European species.

Theory suggests that the stronger the selection of resident alleles over the two sides of a hybrid zone, the narrower the area of the hybrid zone itself (reviewed in [50]). In the absence of recognition mechanisms allowing individuals to mate with conspecifics, hybrid areas can be seen as population sinks and are unlikely to enlarge over larger portions of the areas occupied by the two different entities [53]. So far, no experiments have been conducted on pre- and post-copulatory mechanisms involved in intraspecific mating avoidance in *Zerynthia*, and fitness depression has yet to be demonstrated in *Z. polyxena* and *Z. cassandra* hybrids. In fact, crossing experiments have been performed between *Z. polyxena* and formerly classified *Z. polyxena cassandra* from France [4], which is actually a population of *Z. polyxena.* However, our results suggest that strict recognition systems, strong hybrid depression and/or interactions determining mutual exclusion among these species occur and, as a result, a form of almost complete reproductive barrier emerged in a relatively short evolutionary time. Further investigations may therefore be necessary to clarify the origin of and mechanisms involved in these barriers.

### Distribution Modelling and Conclusive Remarks

The current range of *Z. cassandra* and *Z.polyxena* matched the distribution predicted by climatic models. The only relevant discrepancy between observed and predicted distribution was for central and southern Italy, an area with suitable habitat for *Z. polyxena* but with no records of occurrence. In particular, the model predicted both taxa to be present in Corsica and Sardinia, where no record of them has ever been reported. Similarly, *Z. polyxena* was predicted to occur across most of the Italian Apennines and in Sicily where this species has never been observed.

The projection of climatic models onto glacial maximum scenarios revealed that both species, particularly *Z. cassandra*, probably experienced range contractions which could have favoured isolation and divergence. Modelling of species occurrence resulted in wider distributions recovered by MIROC with respect to CCSM. A similar result was found by [45] and [46]. However, *Z. polyxena* was predicted to occur in the Italian Peninsula during glacial maxima by both models. Therefore neither niche modelling on current distribution nor retrodictions on LGM can explain the absence of *Z. polyxena* from the Italian peninsula.

Morphological, genetic and climatic information and data from other butterfly groups (e.g. [4]) suggest that genitalic divergence and reproductive barriers between *Z. polyxena and Z. cassandra* may have developed in a relatively short time. In fact, most cryptic sister species occurring in Europe show a divergence time similar to that reported for *Z. polyxena* and *Z. cassandra.* However, no instances have been reported of both a strong diversification in genitalic structure and complete absence of introgression, as shown in our study [13], [16], [29], [49]. An important factor determining the evolution of two very distinct morphologies and genetic units is probably due to the low dispersal ability of *Zerynthia* [54]. Moreover, males and females of both species are highly phylopatric and their area of activity mostly depends on the distribution of the same larval host plants (*Aristolochia* spp.) [55]. A good indicator of such a limited dispersal capacity is the absence of both species from most Mediterranean islands, despite suitable climatic conditions and the occurrence of potential host plants. Island populations of *Z. cassandra* are found only on the islands of Elba and Sicily [56], which are both close to the mainland. Similarly, *Z. polyxena* is known to occur on a few islands close to the Balkan peninsula [39]. From this perspective, the presence of two distinct areas with suitable climatic conditions for *Z. polyxena* in northern Italy-France and Greece, respectively, described by the CCSM model for the last glacial maximum, does not necessarily imply the occurrence of this species in both areas. Haplotype networks clearly suggest that both species have strong genetic structure, indicating that several populations might have survived the LGM and previous glacial periods in separate micro-refugia. A prominent pattern in *Z. polyxena*, for instance, is shown by separate groups occurring in eastern Europe (Russia and Romania), northern Italy and France. Tuscany (central Italy) is rich in endemic haplotypes of *Z. cassandra*, suggesting that this region may have functioned as a glacial refugium, as confirmed by distribution modelling projections. Conversely, the area north-west of Tuscany (Liguria and Piemonte) appeared to have been colonized more recently by a single Tuscan haplotype of *Z. cassandra*. *Z. polyxena* from northern Italy showed a more complex situation with most haplotypes being endemic but highly related to the eastern haplogroups and just one haplotype shared with French populations.

Nazari and Sperling [16] estimated a divergence time of 1.8 My (lower Pleistocene) between *Z. polyxena* and *Z. cassandra*, supporting our hypothesis that a series of glacial and interglacial phases could have initiated and maintained the inter and intra-specific diversification process. Our analyses suggest that the last glacial stage did not result in extensive extinctions of *Zerynthia* across the study area and that Italy was probably a suitable habitat for both taxa. Since no relict populations of *Z. polyxena* were found in the Italian peninsula, it is likely that *Z. polyxena* and *Z. cassandra* were allopatric before the LGM, probably as a result of reproductive barriers and/or competition for resources. There is also evidence that *Z. cassandra* came into contact with *Z. polyxena* after a relatively recent colonization event of Liguria and Piemonte from central Italy. This body of evidence suggests that in the last glacial stages *Z. cassandra* probably experienced isolation in several Italian micro-refugia. Such pattern was followed by expansion and marginal contacts with *Z. polyxena* during interglacial periods. The presence of a single haplotype endemic to Elba Island suggests that island colonization may have been hindered by sea level rise at the end of the last Ice Age, while genetic structure of mainland populations changed following complex and recurrent colonization events, as shown for other butterfly species from the same region [57], [58].

Recent literature suggests that speciation can occur even when populations are in contact and able to hybridize (reviewed in [7]). Epistasis conferring enhanced fitness in different areas may result in limited exchange of genomic components associated to reproduction. In *Zerynthia*, genetic interactions affecting shape of genitalia may have played a particular role at the initial stage of diversification, when the two taxa probably came into repeated contact during glacial and interglacial periods. On the other hand, there is evidence that other adaptive and neutral variation may be more easily exchanged among species [6]. In the Mediterranean, distinct species share adaptive variation across the Maghreb and Italy [45], [57], while in Neotropical butterflies adaptive Müllerian wing colouration can be exchanged among species despite their strong genetic diversification [6]. These examples may help explaining why *Z. polyxena* and *Z. cassandra* have maintained identical aposematic wing patterns and colouration [59], [60].

The *Zerynthia* species considered in our study seem to follow the reproductive isolation rule required by the biological species concept. From this perspective, they represent an exception compared to most European sister/cryptic species. We therefore recommend to consider *Z. polyxena* and *Z. cassandra* as good species, also in the restrictive sense of Descimon and Mallet [4], and to promote these taxa as an important model for the study of speciation, correlation between genotypic and phenotypic traits, evolution and maintenance of adaptive patterns.

## Supporting Information

Figure S1 **Schematic representation of fixed landmarks and sliding semi-landmarks considered in geometric morphometric analyses.**
(PDF)

Table S1 Additional information on *Zerynthia polyxena* and *Z. cassandra* samples analysed in this study.
(DOC)

File S1 **Supplementary results for the Maxent model of** ***Z. polyxena.***
(PDF)

File S2 **Supplementary results for the Maxent model of** ***Z. cassandra.***
(PDF)
