# Supplementary material for: When the Rule Becomes the Exception. No Evidence of Gene Flow between Two Zerynthia Cryptic Butterflies Suggests the Emergence of a New Model Group
Source: PLoS One. 2013 Jun 6;8(6):e65746. doi: 10.1371/journal.pone.0065746 (PMC3675026; doi:10.1371/journal.pone.0065746)
Supplement: Figure S1 — Schematic representation of fixed landmarks and sliding semi-landmarks considered in geometric morphometric analyses. (PDF) [file pone.0065746.s001.pdf]

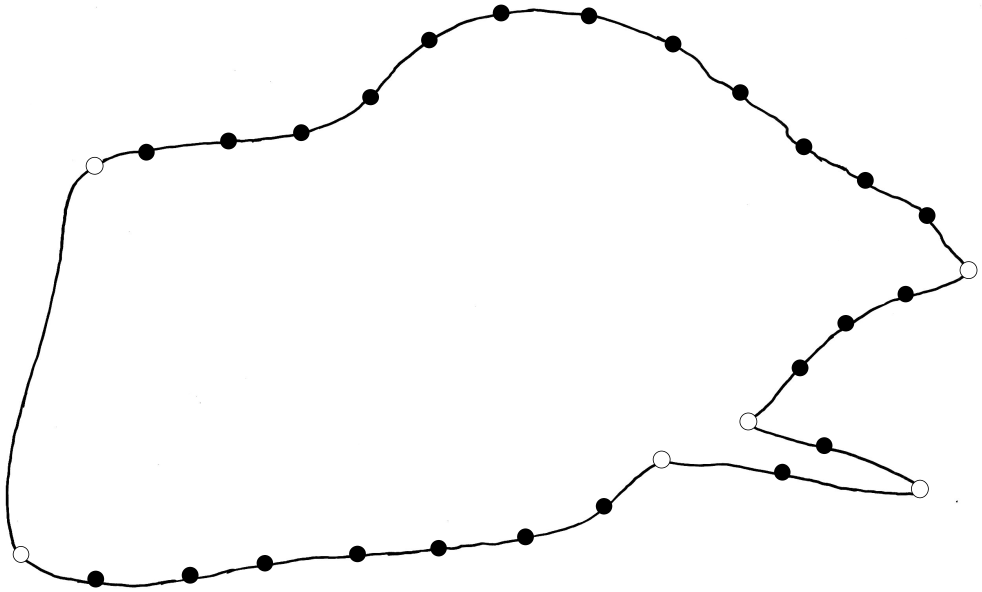

Figure S1. Schematic representation of fixed landmarks (open circles) and sliding semi-landmarks (black circles) considered in geometric morphometric analyses.
