# Supplementary material for: When the Rule Becomes the Exception. No Evidence of Gene Flow between Two Zerynthia Cryptic Butterflies Suggests the Emergence of a New Model Group
Source: PLoS One. 2013 Jun 6;8(6):e65746. doi: 10.1371/journal.pone.0065746 (PMC3675026; doi:10.1371/journal.pone.0065746)
Supplement: Table S1 — Additional information on Zerynthia polyxena and Z. cassandra samples analysed in this study. (DOC) [file pone.0065746.s002.doc]

**Table S1** Additional information on *Zerynthia polyxena* and *Z. cassandra* samples analysed in this study. Hyphens denote missing information of either genetic or morphologial markers. MZUF: Natural History Museum of the University of Florence; IBE: Institute of Evolutionary Biology, Barcelona, ZLT: Zoology Laboratory, University of Turin.

| **Sample ID** | **Museum collection** | **Taxon** | **COI haplotype** | **ND1 haplotype** | **wg genotype** | **Genitalia morphotype** | **Sampling location** | **Collector** |
| --- | --- | --- | --- | --- | --- | --- | --- | --- |
| LD1031 | MZUF | *Z. polyxena* | C16 | - | W2 | *polyxena* | France, Sollies Pont, Var | Eric Bonora |
| LD1032 | MZUF | *Z. polyxena* | C17 | - | W2 | *polyxena* | France, Sollies Pont, Var | Eric Bonora |
| LD1033 | MZUF | *Z. polyxena* | C18 | N11 | W2 | *polyxena* | France, Sollies Pont, Var | Eric Bonora |
| LD1034 | MZUF | *Z. polyxena* | C19 | - | - | *polyxena* | France, Sollies Pont, Var | Eric Bonora |
| LD1063 | MZUF | *Z. polyxena* | C21 | N12 | W2 | *polyxena* | France, Hautes Alpes St Crepin | Eric Bonora |
| LD1065 | MZUF | *Z. polyxena* | C22 | N11 | W2 | *polyxena* | France, Hautes Alpes St Crepin | Eric Bonora |
| LD1020 | MZUF | *Z. polyxena* | C13 | - | W4 | *polyxena* | Italy, Salbertrand (TO) | Eric Bonora |
| LD1021 | MZUF | *Z. polyxena* | C13 | - | W2 | *polyxena* | Italy, Salbertrand (TO) | Eric Bonora |
| LD1025 | MZUF | *Z. polyxena* | C13 | N9 | W3 | *polyxena* | Italy, Salbertrand (TO) | Eric Bonora |
| LD1026 | MZUF | *Z. polyxena* | C14 | N9 | W2 | *polyxena* | Italy, Salbertrand (TO) | Eric Bonora |
| LD1027 | MZUF | *Z. polyxena* | C13 | N10 | W4 | *polyxena* | Italy, Salbertrand (TO) | Eric Bonora |
| LD1028 | MZUF | *Z. polyxena* | C15 | - | W2 | *polyxena* | Italy, Salbertrand (TO) | Eric Bonora |
| LD1030 | MZUF | *Z. polyxena* | C14 | - | - | - | Italy, Salbertrand (TO) | Eric Bonora |
| LD1053 | MZUF | *Z. polyxena* | C13 | - | W2 | *polyxena* | Italy, Val della Torre (TO) | Eric Bonora |
| LD1054 | MZUF | *Z. polyxena* | C13 | N10 | W2 | *polyxena* | Italy, Val della Torre (TO) | Eric Bonora |
| LD1036 | MZUF | *Z. polyxena* | C13 | N9 | W4 | *polyxena* | Italy, Greggio (VC) | Eric Bonora |
| LD1037 | MZUF | *Z. polyxena* | C13 | - | W2 | *polyxena* | Italy, Greggio (VC) | Eric Bonora |
| LD1038 | MZUF | *Z. polyxena* | C13 | N9 | W2 | *polyxena* | Italy, Greggio (VC) | Eric Bonora |
| LD1040 | MZUF | *Z. polyxena* | C20 | N11 | W2 | *polyxena* | Italy, Greggio (VC) | Eric Bonora |
| LD1018 | MZUF | *Z. polyxena* | C13 | - | W2 | *polyxena* | Italy, Vigevano (PV) | Eric Bonora |
| LDA1 | MZUF | *Z. polyxena* | C13 | N10 | W2 | *polyxena* | Italy, Alpicella, Mount Beigua (SV) | Enrico Punta |
| SV3 | MZUF | *Z. polyxena* | C13 | - | W2 | *polyxena* | Italy, Alpicella, Mount Beigua (SV) | Enrico Punta |
| SV10 | MZUF | *Z. polyxena* | C13 | - | W2 | *polyxena* | Italy, Alpicella, Mount Beigua (SV) | Enrico Punta |
| IV3 | ZLT | *Z. polyxena* | C14 | - | - | - | Italy, Ivrea (TO) | Authors |
| C4 | ZLT | *Z. cassandra* | C1 | N1 | - | *cassandra* | Italy, Castelnuovo (AL) | Authors |
| C5 | ZLT | *Z. cassandra* | C1 | N1 | W1 | *cassandra* | Italy, Castelnuovo (AL) | Authors |
| C6 | ZLT | *Z. cassandra* | C1 | N1 | - | - | Italy, Castelnuovo (AL) | Authors |
| C7 | ZLT | *Z. cassandra* | C1 | N1 | W1 | *cassandra* | Italy, Castelnuovo (AL) | Authors |
| C8 | ZLT | *Z. cassandra* | - | N1 | W1 | - | Italy, Castelnuovo (AL) | Authors |
| M10 | ZLT | *Z. cassandra* | C1 | N13 | W1 | *cassandra* | Italy, Marcarolo (AL) | Authors |
| M11 | ZLT | *Z. cassandra* | C1 | N14 | W1 | *cassandra* | Italy, Marcarolo (AL) | Authors |
| LDA2 | MZUF | *Z. cassandra* | C1 | N1 | W1 | *cassandra* | Italy, Alpicella, Mount Beigua (SV) | Enrico Punta |
| LDA3 | MZUF | *Z. cassandra* | C1 | N1 | W1 | - | Italy, Alpicella, Mount Beigua (SV) | Enrico Punta |
| LD3386 | MZUF | *Z. cassandra* | - | N1 | - | - | Italy, Alpicella, Mount Beigua (SV) | Enrico Punta |
| LD3387 | MZUF | *Z. cassandra* | - | N1 | - | - | Italy, Alpicella, Mount Beigua (SV) | Enrico Punta |
| LD3388 | MZUF | *Z. cassandra* | C1 | N1 | W1 | *cassandra* | Italy, Alpicella, Mount Beigua (SV) | Enrico Punta |
| SV4 | MZUF | *Z. cassandra* | C1 | - | W1 | *cassandra* | Italy, Alpicella, Mount Beigua (SV) | Enrico Punta |
| SV5 | MZUF | *Z. cassandra* | C1 | - | W1 | *cassandra* | Italy, Alpicella, Mount Beigua (SV) | Enrico Punta |
| SP6 | MZUF | *Z. cassandra* | C1 | - | W1 | *cassandra* | Italy, Alpicella, Mount Beigua (SV) | Enrico Punta |
| SV8 | MZUF | *Z. cassandra* | C1 | - | W1 | *cassandra* | Italy, Alpicella, Mount Beigua (SV) | Enrico Punta |
| LD3166 | IBE | *Z. cassandra* | C1 | N1 | W1 | *cassandra* | Italy, San Martino-Stella (SV) | Authors |
| LD3167 | IBE | *Z. cassandra* | C1 | N1 | W1 | *cassandra* | Italy, San Martino-Stella (SV) | Authors |
| LD3168 | IBE | *Z. cassandra* | C1 | N1 | W1 | *cassandra* | Italy, San Martino-Stella (SV) | Authors |
| LD3170 | IBE | *Z. cassandra* | C1 | N1 | W1 | *cassandra* | Italy, San Martino-Stella (SV) | Authors |
| LD3171 | IBE | *Z. cassandra* | C1 | N1 | W1 | *cassandra* | Italy, San Martino-Stella (SV) | Authors |
| LD1010 | MZUF | *Z. cassandra* | C1 | N1 | W1 | *cassandra* | Italy, Brescello (RE) | Authors |
| LD1011 | MZUF | *Z. cassandra* | C1 | N1 | W1 | *cassandra* | Italy, Brescello (RE) | Authors |
| LD1012 | MZUF | *Z. cassandra* | C1 | N1 | W1 | *cassandra* | Italy, Brescello (RE) | Authors |
| LD1013 | MZUF | *Z. cassandra* | C1 | N1 | W1 | - | Italy, Brescello (RE) | Authors |
| LD19 | MZUF | *Z. cassandra* | C3 | N2 | W1 | *cassandra* | Italy, San Rossore (PI) | Authors |
| LD1045 | MZUF | *Z. cassandra* | C1 | N1 | W1 | *cassandra* | Italy, Marina di Vecchiano (PI) | Authors |
| LD960 | IBE | *Z. cassandra* | C2 | N1 | W1 | *cassandra* | Italy, Elba Island (LI) | Authors |
| LD961 | IBE | *Z. cassandra* | C2 | N1 | W1 | *cassandra* | Italy, Elba Island (LI) | Authors |
| LD962 | IBE | *Z. cassandra* | C2 | N1 | W1 | - | Italy, Elba Island (LI) | Authors |
| LD964 | IBE | *Z. cassandra* | C2 | N1 | W1 | *cassandra* | Italy, Elba Island (LI) | Authors |
| LD965 | IBE | *Z. cassandra* | C2 | N1 | W1 | *cassandra* | Italy, Elba Island (LI) | Authors |
| LD966 | IBE | *Z. cassandra* | C2 | N1 | W1 | - | Italy, Elba Island (LI) | Authors |
| LD968 | IBE | *Z. cassandra* | C2 | N1 | W1 | - | Italy, Elba Island (LI) | Authors |
| LD969 | IBE | *Z. cassandra* | C2 | N1 | W1 | *cassandra* | Italy, Elba Island (LI) | Authors |
| LD970 | IBE | *Z. cassandra* | C2 | N1 | W1 | - | Italy, Elba Island (LI) | Authors |
| LD971 | IBE | *Z. cassandra* | C2 | N1 | W1 | - | Italy, Elba Island (LI) | Authors |
| LD1044 | IBE | *Z. cassandra* | C2 | N1 | W1 | - | Italy, Elba Island (LI) | Authors |
| LD978 | MZUF | *Z. cassandra* | C3 | N2 | W1 | *cassandra* | Italy, Prato-San Giorgio (PO) | Authors |
| LD983 | MZUF | *Z. cassandra* | C3 | N2 | W1 | *cassandra* | Italy, Prato-San Giorgio (PO) | Authors |
| LD986 | MZUF | *Z. cassandra* | C4 | N1 | W1 | *cassandra* | Italy, Prato-San Giorgio (PO) | Authors |
| LD987 | MZUF | *Z. cassandra* | C3 | N2 | W1 | *cassandra* | Italy, Prato-San Giorgio (PO) | Authors |
| LD989 | MZUF | *Z. cassandra* | C3 | N2 | W1 | *cassandra* | Italy, Prato-San Giorgio (PO) | Authors |
| LD1002 | MZUF | *Z. cassandra* | C3 | N2 | W1 | *cassandra* | Italy, Prato-San Giorgio (PO) | Authors |
| LD1003 | MZUF | *Z. cassandra* | C4 | N1 | W1 | - | Italy, Prato-San Giorgio (PO) | Authors |
| LD1006 | MZUF | *Z. cassandra* | C1 | N1 | W1 | *cassandra* | Italy, Prato-San Giorgio (PO) | Authors |
| LD1043 | MZUF | *Z. cassandra* | C5 | N2 | W1 | *cassandra* | Italy, Prato-Calvana (PO) | Authors |
| LD3351 | MZUF | *Z. cassandra* | C10 | N5 | W1 | - | Italy, Grecciano (LI) | Eric Bonora |
| G1 | MZUF | *Z. cassandra* | C3 | N2 | W1 | *cassandra* | Italy, Grecciano (LI) | Eric Bonora |
| G2 | MZUF | *Z. cassandra* | C3 | N2 | W1 | *cassandra* | Italy, Grecciano (LI) | Eric Bonora |
| G3 | MZUF | *Z. cassandra* | C1 | N1 | W1 | *cassandra* | Italy, Grecciano (LI) | Eric Bonora |
| G4 | MZUF | *Z. cassandra* | C3 | N2 | W1 | *cassandra* | Italy, Grecciano (LI) | Eric Bonora |
| LDL1 | MZUF | *Z. cassandra* | C1 | - | W1 | - | Italy; Grosseto (GR) | Authors |
| LDL2 | MZUF | *Z. cassandra* | C1 | N1 | W1 | - | Italy; Grosseto (GR) | Authors |
| LDL4 | MZUF | *Z. cassandra* | C1 | N1 | W1 | - | Italy; Grosseto (GR) | Authors |
| LDL5 | MZUF | *Z. cassandra* | C1 | N1 | W1 | - | Italy; Grosseto (GR) | Authors |
| LDL6 | MZUF | *Z. cassandra* | C1 | N1 | W1 | - | Italy; Grosseto (GR) | Authors |
| LDL7 | MZUF | *Z. cassandra* | C1 | N1 | W1 | - | Italy; Grosseto (GR) | Authors |
| LDL8 | MZUF | *Z. cassandra* | C1 | N1 | W1 | - | Italy; Grosseto (GR) | Authors |
| LDL9 | MZUF | *Z. cassandra* | C1 | N1 | W1 | - | Italy; Grosseto (GR) | Authors |
| LD1866 | IBE | *Z. cassandra* | C2 | N2 | W1 | *cassandra* | Italy, Sansa (SA) | Authors |
| LD1867 | IBE | *Z. cassandra* | C1 | N2 | W1 | *cassandra* | Italy, Sansa (SA) | Authors |
| LD1868 | IBE | *Z. cassandra* | C1 | N2 | W1 | *cassandra* | Italy, Sansa (SA) | Authors |
| LD1869 | IBE | *Z. cassandra* | C1 | N2 | W1 | - | Italy, Sansa (SA) | Authors |
| LD1870 | IBE | *Z. cassandra* | C1 | N2 | W1 | - | Italy, Sansa (SA) | Authors |
| LD1871 | IBE | *Z. cassandra* | C1 | N2 | W1 | - | Italy, Sansa (SA) | Authors |
| LD1873 | IBE | *Z. cassandra* | C1 | N2 | W1 | *cassandra* | Italy, Sansa (SA) | Authors |
| LD1874 | IBE | *Z. cassandra* | C1 | N2 | W1 | *cassandra* | Italy, Sansa (SA) | Authors |
| LD1875 | IBE | *Z. cassandra* | C2 | N3 | W1 | *cassandra* | Italy, Sansa (SA) | Authors |
| LDASP1 | IBE | *Z. cassandra* | C11 | N6 | W1 | - | Italy, Aspromonte (RC) | Authors |
| LDASP2 | IBE | *Z. cassandra* | C12 | N7 | W1 | - | Italy, Aspromonte (RC) | Authors |
| LD3130 | IBE | *Z. cassandra* | C6 | N4 | W1 | *cassandra* | Italy, Sicily Island, Etna, Ragalna (CT) | Authors |
| LD3131 | IBE | *Z. cassandra* | C2 | N4 | W1 | *cassandra* | Italy, Sicily Island, Etna, Ragalna (CT) | Authors |
| LD3132 | IBE | *Z. cassandra* | C8 | N2 | W1 | *cassandra* | Italy, Sicily Island, Etna, Ragalna (CT) | Authors |
| LD3133 | IBE | *Z. cassandra* | C9 | N2 | W1 | *cassandra* | Italy, Sicily Island, Etna, Ragalna (CT) | Authors |
| LD3134 | IBE | *Z. cassandra* | C6 | N4 | W1 | *cassandra* | Italy, Sicily Island, Etna, Ragalna (CT) | Authors |
| LD3135 | IBE | *Z. cassandra* | C6 | N2 | W1 | *cassandra* | Italy, Sicily Island, Etna, Ragalna (CT) | Authors |
| RV11-D290 | IBE | *Z. cassandra* | C8 | - | - | - | Italy, Sicily Island, Etna, Ragalna (CT) | Authors |
| RV11-I061 | IBE | *Z. cassandra* | C6 | - | - | - | Italy, Sicily Island, Randazzo (CT) | Authors |
| LDET1 | IBE | *Z. cassandra* | C6 | N8 | W1 | - | Italy, Sicily Island, Etna (CT) | Authors |
| LD3128 | IBE | *Z. cassandra* | C6 | N2 | W1 | *cassandra* | Italy, Sicily Island, Monte Busambra (PA) | Authors |
| LD3129 | IBE | *Z. cassandra* | C7 | N2 | W1 | *cassandra* | Italy, Sicily Island, Monte Busambra (PA) | Authors |
