# Supplementary material for: When the Rule Becomes the Exception. No Evidence of Gene Flow between Two Zerynthia Cryptic Butterflies Suggests the Emergence of a New Model Group
Source: PLoS One. 2013 Jun 6;8(6):e65746. doi: 10.1371/journal.pone.0065746 (PMC3675026; doi:10.1371/journal.pone.0065746)
Supplement: File S1 — Supplementary results for the Maxent model of Z. polyxena. (PDF) [file pone.0065746.s003.pdf]

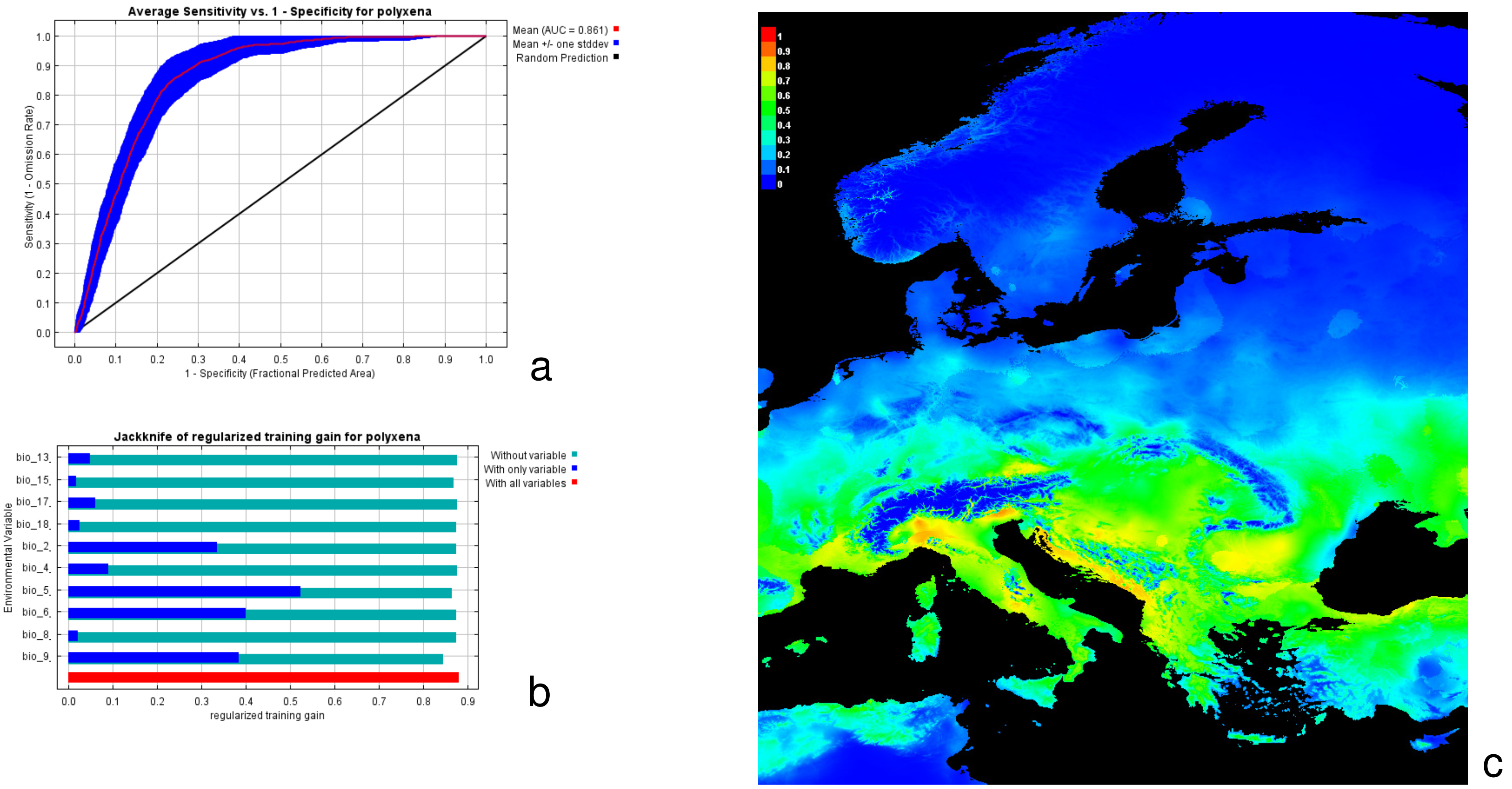

File S1. Supplementary results for the Maxent model of *Z. polyxena*, ROC curve (a); jackknife evaluation for the relative importance of BIOCLIM variables (b); logistic output for the entire study area (c).
